# Multimodal bipedal locomotion generation with passive dynamics *via* deep reinforcement learning

**DOI:** 10.3389/fnbot.2022.1054239

**Published:** 2023-01-23

**Authors:** Shunsuke Koseki, Kyo Kutsuzawa, Dai Owaki, Mitsuhiro Hayashibe

**Affiliations:** Neuro-Robotics Lab, Department of Robotics, Graduate School of Engineering, Tohoku University, Sendai, Japan

**Keywords:** bipedal walking and running, gait transition, deep reinforcement learning, underactuated robot, embodiment

## Abstract

Generating multimodal locomotion in underactuated bipedal robots requires control solutions that can facilitate motion patterns for drastically different dynamical modes, which is an extremely challenging problem in locomotion-learning tasks. Also, in such multimodal locomotion, utilizing body morphology is important because it leads to energy-efficient locomotion. This study provides a framework that reproduces multimodal bipedal locomotion using passive dynamics through deep reinforcement learning (DRL). An underactuated bipedal model was developed based on a passive walker, and a controller was designed using DRL. By carefully planning the weight parameter settings of the DRL reward function during the learning process based on a curriculum learning method, the bipedal model successfully learned to walk, run, and perform gait transitions by adjusting only one command input. These results indicate that DRL can be applied to generate various gaits with the effective use of passive dynamics.

## 1. Introduction

Humans exhibit multimodal gait patterns such as walking, running, skipping, and jumping (Diedrich and Warren, 1995; Alexander, 1996; Minetti and Alexander, 1997). Moreover, the gait transition between walking and running is observed at the speed boundaries, known as the preferred transition speed (PTS) (Sharbafi and Seyfarth, 2017). With the PTS as the boundary, walking at low speeds and running at high speeds are gaits with optimal energy efficiency in each speed domain (Diedrich and Warren, 1995; Alexander, 1996; Minetti and Alexander, 1997; Srinivasan and Ruina, 2006; Sharbafi and Seyfarth, 2017). To achieve such situation-dependent multimodal bipedal locomotion, not only neural control systems but also body morphology plays a crucial role based on the concept of *embodiment* (Pfeifer and Scheier, 2001; Owaki et al., 2008). Reproducing such human multimodal behaviors in a robot can broaden its locomotion ability as well as improve the understanding of the underlying mechanisms of human gaits and their transitions.

Despite the overwhelming complexity of their inherent dynamics, human locomotion can be represented as a simple conceptual model (Sharbafi and Seyfarth, 2017). Specifically, human walking can be represented as an inverted pendulum (IP) model (Kuo, 2007). During walking, the stance leg behaves as an inverted pendulum that rotates around the ankle joint. Additionally, the changes in the kinematic and potential energies are out-of-phase (i.e., when one is at its positive peak, the other is at its negative peak), whereas the mechanical energy remains almost constant (Cavagna et al., 1976, 2000; Cavagna and Legramandi, 2020). In contrast, the body dynamics of human running are different in that the changes in the kinematic and potential energies are in-phase (i.e., they reach their corresponding phases at the same time) (Cavagna et al., 1976; Cavagna, 2006). When both the kinematic and potential energies decrease, some amount of energy is stored as elastic energy in the spring-like elements of the body, such as muscles, tendons, and ligaments (Farley and Gonzalez, 1996). Inspired by such biomechanical processes, the spring-loaded inverted pendulum (SLIP) model (Blickhan, 1989; Dickinson et al., 2000) has been adopted to explain and analyze running.

Passive dynamic walking is also based on the inverted pendulum mechanism (McGeer, 1990). In passive walking, a bipedal machine can walk down a gentle slope stably by using only its body dynamics without any actuators. The behavior is purely generated through the interaction between its body and the environment, and the control system is not involved in it. By generating motions exploiting its body morphology, a certain amount of computation for generating the behaviors can be *offloaded* to the body (Owaki et al., 2008; Pfeifer and Gómez, 2009). This reduces the computational cost to the control system and leads to energy-efficient locomotion (Collins and Ruina, 2005; Pfeifer and Gómez, 2009; Bhounsule et al., 2012). Thus, to realize these benefits, it is necessary to use passive dynamics and exploit the body morphology to generate movements.

However, despite the importance of passive dynamics in bipedal locomotion control, it has not been sufficiently investigated in previous bipedal walking and running robots (Hodgins, 1991; Kwon and Park, 2003; Nagasaka et al., 2004; Tajima et al., 2009; Sreenath et al., 2013; Kobayashi et al., 2016; Siekmann et al., 2021). There are two possible reasons for this: (1) Robots with passive joints are more difficult to control than fully actuated robots because there is less scope for the control system to intervene in motion generation. (2) As previously mentioned, walking and running are dynamically different locomotion modes; hence, it is difficult to reproduce multimodal locomotion from a single controller (Smit-Anseeuw et al., 2017; Okajima et al., 2018). Because body dynamics is partially determined by passive joints, generating dynamically different locomotion modes using passive dynamics is a challenging problem.

To address this issue, this study utilized deep reinforcement learning (DRL). In recent years, DRL has attracted attention as a promising technique for generating gaits in robotic systems. The advantage of DRL is that it learns locomotion skills with minimal craftsmanship and does not require careful modeling of the robot dynamics (Haarnoja et al., 2018a; Hwangbo et al., 2019). Previous studies have demonstrated that DRL can acquire controllers for multimodal gait in legged robots. Siekmann et al. (2021) presented a reward specification framework and demonstrated multimodal gaits in bipedal robots, including walking, running, hopping, and their transitions without prior knowledge. Fu et al. (2021) proposed a method for generating the walking, trotting, and bouncing gaits, and achieved smooth gait transitions in a quadrupedal robot by using a single controller *via* a stage-wise distillation approach. Shao et al. (2021) used imitation learning with the guided phase generated by the central pattern generator on a quadruped robot, and demonstrated multiple gaits and smooth transitions. Moreover, DRL can facilitate the realization of controllers for challenging locomotion skills in bipedal robots. Xie et al. (2020) presented a general learning scheme for navigating stepping stones. The bipedal robots in the simulation environment succeeded in walking on terrains consisting of discrete foot placements without falling or stopping. Yu et al. (2018) demonstrated realistic and smooth walking and running through simulation even though they did not make use of prior knowledge for training. In addition, the controllers generated through DRL are robust to variations in system dynamics, such as sensory delays, uneven terrain, and blind conditions (Xie et al., 2018; Castillo et al., 2021; Kang and Lee, 2021; Li et al., 2021).

The purpose of this study is to generate multimodal gaits such as walking, running, and their transitions using passive dynamics through DRL. For this, a bipedal model based on a passive walker was developed using numerical simulation. Subsequently, a reward function and learning scheme was designed for the DRL. The trained controller could achieve walking, running, and their transitions by adjusting only one input command. This study makes two significant contributions to the state of the art. The first contribution is that it established DRL as a promising technique for generating multimodal gaits using passive dynamics. The second contribution is that it presents a learning framework for training a simple control policy for a bipedal robot to switch between walking and running based on only a speed parameter without reference motion.

## 2. Methods

### 2.1. Bipedal model

**Figure 1:**
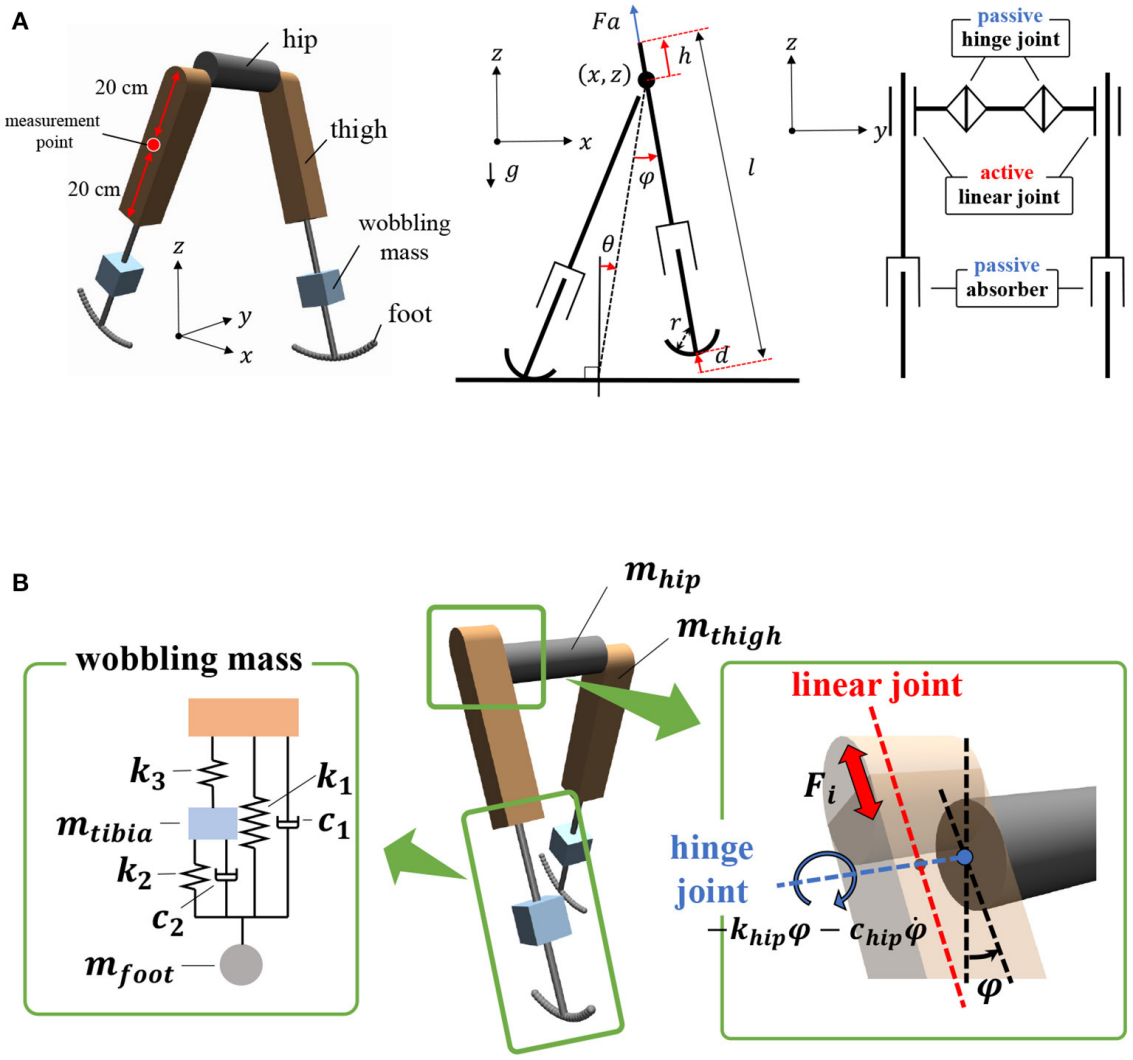
Structure of the bipedal model. The motions are constrained in the *x*–*z* plane, i.e., the sagittal plane. **(A)** Left: Oblique view of the bipedal model in MuJoCo simulator. Center: Side view of mechanical structure of the model. Right: Front view of mechanical structure. **(B)** Left: Schematic of wobbling mass mechanism. Right: Active linear actuators and passive rotational springs were implemented to thigh segments and hip joints, respectively.

The bipedal model employed in this study is shown in Figure 1. The model parameters are presented in Table 1. The basic mechanical structure is based on the passive dynamic bipedal model from our previous studies (Owaki et al., 2008, 2011). Motion is constrained in the sagittal plane. Notably, the hip joints are passive, i.e., they do not have actuators. Thus, the model needs to indirectly control hip joints such that the controller effectively exploits its body dynamics to swing its legs and move forward. Each leg has a linear actuator with a maximum magnitude of force *F* that moves the thigh segment up and down along the leg axis (red dotted line in Figure 1B). These actuators can push off the ground to generate a propulsion force in the stance phase and lift the corresponding leg to generate ground clearance of the foot in the swing phase. The hip joints (blue dotted line in Figure 1B) are passive rotational joints with torsional springs (*k*_*hip*_) and dampers (*c*_*hip*_). The spring generates rotational forces to swing the leg forward and backward, and prevents it from opening too widely (Owaki et al., 2011). In addition, a wobbling mass mechanism (Yue and Mester, 2002; Nikooyan and Zadpoor, 2011) consisting of three linear springs (*k*_1_, *k*_2_, *k*_3_), two dampers (*c*_1_, *c*_2_), and a mass (*m*_*tibia*_) was employed, as shown in Figure 1B. This mechanism contributes to reducing the impact between the foot and ground during foot–ground contact. The model has arc-shaped rigid feet with radius *r* = *l*/3 (Hansen et al., 2004), where *l* is the leg length. Each foot in the model consists of 20 small spheres. The state variables of the model are the hip segment positions *x* and *z*, hip segment orientation θ, leg angles φ_*j*_, displacements of the thigh segments *h*_*j*_, contractions of legs *d*_*j*_, and their time derivatives. Here, suffix *j* denotes the leg (*j* = *r*: right and *j* = *l*: left).

**Table 1:** Parameters of the bipedal model.

**Parameter**	**Unit**	**Value**	**Parameter**	**Unit**	**Value**
*l*	[m]	0.8	*k*_*hip*_	[Nm/rad]	25
*r*	[m]	0.27	*c*_*hip*_	[Nms/rad]	2
*m*_*hip*_	[kg]	20	*k*_1_	[N/m]	6,000
*m*_*thigh*_	[kg]	6	*k*_2_	[N/m]	6,000
*m*_*tibia*_	[kg]	3	*k*_3_	[N/m]	10,000
*m*_*foot*_	[kg]	1	*c*_1_	[Ns/m]	300
			*c*_2_	[Ns/m]	650
			*F*	[N]	600

### 2.2. Deep reinforcement learning

**Figure 2:**
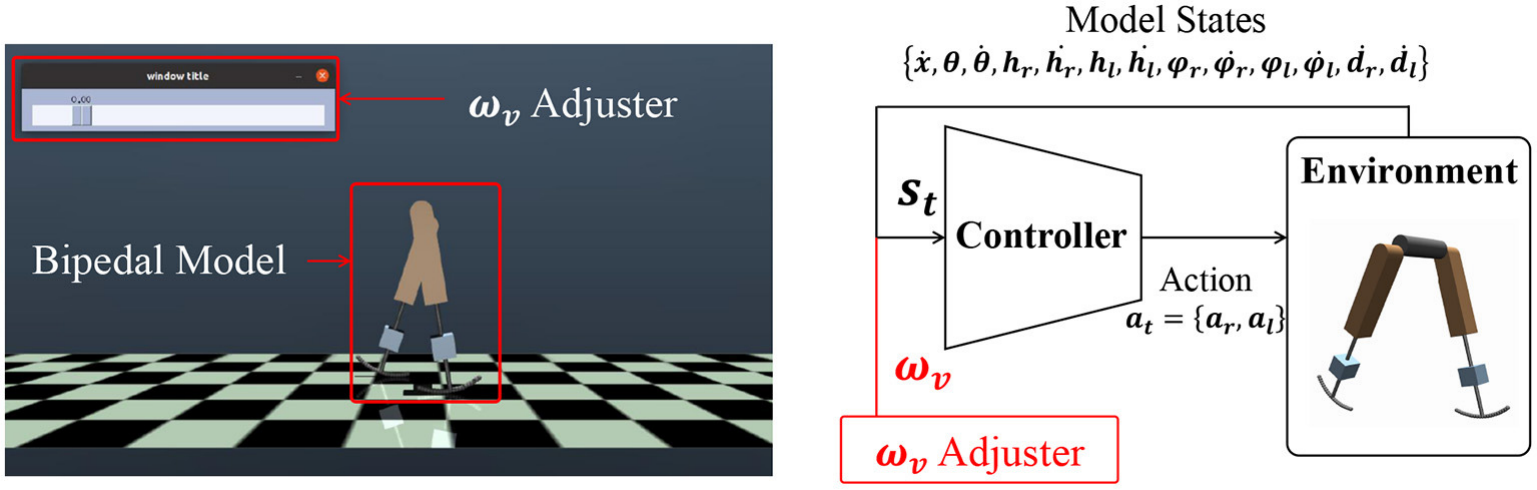
Bipedal model control in a simulation environment and the basic diagram of the control system. The inputs for the controller include a command ω_*v*_. The value of ω_*v*_ can be changed through the ω_*v*_ adjuster. *s*_*t*_ consists of the model states ($ẋ,\theta ,\overset{∙}{\theta },h_{r},{ḣ}_{r},h_{l},{ḣ}_{l},\varphi_{r},{\overset{∙}{\varphi }}_{r},\varphi_{l},{\overset{∙}{\varphi }}_{l},{ḋ}_{r},{ḋ}_{l}$) and ω_*v*_.

In this study, we designed a controller that outputs actuator signals for the input model states and a speed command, as shown in Figure 2. This system diagram can be seen in a previous study (Saputra et al., 2020). The controller was trained through deep reinforcement learning (DRL). DRL learns an action that maximizes the expected cumulative reward for the observed state through numerous trial-and-error iterations. This study adopted a soft actor-critic (SAC) (Haarnoja et al., 2018b), which is a model-free DRL algorithm for continuous control tasks, because it is the state-of-the-art technique and is better in terms of exploration. In this algorithm, a bonus reward α*H*(π) is added, where *H*(π) is the entropy of the policy π. This term improves the exploration and provides robustness to policies (Haarnoja et al., 2018b). A stochastic policy π is obtained to maximize the objective function *J*(π):


(1) $$\begin{array}{l} J\left(\pi \right)=\overset{T}{\underset{t=0}{\sum }}E_{\left(s_{t},a_{t}\right)\sim \rho_{\pi }}\left[\gamma^{t}\left(r\left(s_{t},a_{t}\right)+\alpha H\left(\pi \left(\cdot |s_{t}\right)\right)\right)\right], \end{array}$$

where γ is the discount rate, *r* denotes the reward function (described in detail later), and α is the temperature parameter, which determines the emphasis of the entropy term. *s*_*t*_ and *a*_*t*_ denote the states and actions, respectively. In this study, $s_{t}\in {\mathbb{R}}^{14}$ comprises model states and a command parameter ω_*v*_ (details of which are provided in Section 2.3.2), and $a_{t}\in {\mathbb{R}}^{2}$ comprises the linear actuator signals *a*_*r*_∈[−1, 1] and *a*_*l*_∈[−1, 1]. Each actuator produces a force *a*_*j*_*F* (Figure 1A).

### 2.3. Learning methods

The objective is to realize a single controller that can achieve walking, running, and their transitions. In this section, we propose a learning framework for realizing a controller that generates multimodal gaits according to an input speed command ω_*v*_, as shown in Figure 2, without any reference motions. We explain the design of the reward function and the learning scheme for DRL.

#### 2.3.1. Design of reward function

In this study, the following reward function was used:


(2) $$\begin{array}{l} r\left(s_{t},a_{t}\right)=\frac{1}{1+\omega_{v}}\left(-\omega_{E}|E_{t}-E_{t-1}|+\omega_{v}ẋ+f_{forward}+f_{alive}+f_{support}\right), \end{array}$$

(3) $$f_{forward}=\{\begin{array}{ll} 0 & \left(\dot{x}\geq 0\right)\\ -C_{1} & \left(\dot{x}<0\right) \end{array},$$

(4) $$\begin{array}{l} f_{alive}=C_{2}, \end{array}$$

Where ω_*i*_ represents the weight coefficients used to determine the relative significance of each term, *E*_*t*_ is the total energy, i.e., the sum of the potential, kinematic, and elastic energies of the model in *t* steps, ẋ is the velocity of the hip segment in the horizontal direction, and *C*_1_ and *C*_2_ are constant values. The first term −ω_*E*_|*E*_*t*_−*E*_*t*−1_| represents the penalty for the total energy variation. When the actuator injects excessive energy into the model, or when the energy lost at touchdown is significant, this term assigns a negative reward. The second term ω_*v*_ẋ is the reward for the forward velocity of a bipedal robot. The term *f*_*forward*_ represents a reward for maintaining the model in forward motion, where a large negative constant *C*_1_ is added if the model does not move forward after a step. As presented later, the weight coefficient of the velocity term varied as ω_*v*_∈[0, 2.5] during the training. We set *f*_*forward*_ because when ω_*v*_ = 0, there is no factor to determine the movement direction in the reward function. The term *f*_*alive*_ prevents the model from falling over. The episode is terminated if |θ|>1.4rad, i.e., the model tilts by more than 80°, even if the maximum length of the step for an episode is not reached. Therefore, if the model does not fall over, this term will always provide a positive reward, and the agent can obtain a larger cumulative reward. The term *f*_*support*_ enables more efficient learning by adding other rewards during the learning process as follows:


(5) $$\begin{array}{l} \begin{array}{c} f_{support}=\omega_{l}f_{leg}+\omega_{s}f_{sym}, \end{array} \end{array}$$

(6) $$\begin{array}{l} f_{leg}=\min\left(|{\overset{{}^{\circ}}{\varphi }}_{r}-{\overset{{}^{\circ}}{\varphi }}_{l}|,C_{3}\right), \end{array}$$

(7) $$\begin{array}{l} f_{sym}=-|\pi \left(s_{t}\right)-{\text{Ψ}}_{a}\left(\pi \left({\text{Ψ}}_{o}\left(s_{t}\right)\right)\right){|}^{2}, \end{array}$$

where the term *f*_*leg*_ enables the legs to swing effectively, and ${\overset{∙}{\varphi }}_{r}-{\overset{∙}{\varphi }}_{l}$ is the time derivative of the angle between the right and left legs (see in Figure 1A). *C*_3_ is a constant with an upper limit to prevent an excessive outward swing of the legs. Because the hip joints are passive and cannot be driven directly, the agent had difficulty in finding the movement to swing the legs. *f*_*leg*_ encourages the policy to swing the legs and leads to efficient exploration. The term *f*_*sym*_ (Yu et al., 2018) introduces symmetry to the policy for movement generation to reproduce human-like symmetric movements during normal walking and running. Ψ_*a*_ and Ψ_*o*_ are functions that map the actions *a*_*t*_ and *s*_*t*_, respectively, into their mirrored versions. As we will see later, the input for the policy *s*_*t*_ is described as $s_{t}=\left\{ẋ,\theta ,\overset{∙}{\theta },h_{r},{ḣ}_{r},h_{l},{ḣ}_{l},\varphi_{r},{\overset{∙}{\varphi }}_{r},\varphi_{l},{\overset{∙}{\varphi }}_{l},{ḋ}_{r},{ḋ}_{l},\omega_{v}\right\}$. Ψ_*o*_ maps *s*_*t*_ to the state $s_{t}^{mirror}$, where the right and left legs are interchanged. $s_{t}^{mirror}={\text{Ψ}}_{o}\left(s_{t}\right)=\left\{ẋ,\theta ,\overset{∙}{\theta },h_{l},{ḣ}_{l},h_{r},{ḣ}_{r},\varphi_{l},{\overset{∙}{\varphi }}_{l},\varphi_{r},{\overset{∙}{\varphi }}_{r},{ḋ}_{l},{ḋ}_{r},\omega_{v}\right\}$. Ψ_*a*_ swaps the values of the right and left leg actuator outputs. Ψ_*a*_(*a*_*t*_) = Ψ_*a*_({*a*_*right*_, *a*_*left*_}) = {*a*_*left*_, *a*_*right*_}. *f*_*sym*_ penalizes the square deviation between the current state and mirrored state (for details, see Yu et al., 2018). 1/(1+ω_*v*_) was set to reduce the inter-reward variability. The reward function calculates widely different values depending on the given variable ω_*v*_∈[0, 2.5]. Assuming that the bipedal model moved to 2.5m/s, if 1/(1+ω_*v*_) is not included, the agent is rewarded up to 1.6 for ω_*v*_ = 0 and 7.85 for ω_*v*_ = 2.5. If 1/(1+ω_*v*_) is included, the agent is rewarded up to 1.6 for ω_*v*_ = 0 and 2.25 for ω_*v*_ = 2.5. Thus, we can reduce the variance among the rewards. The ranges of ω_*v*_ and constant values ω_*E*_, ω_*l*_, ω_*s*_, *C*_1_, *C*_2_, and *C*_3_ were set as shown in Table 2 through trial and error.

**Table 2:** Parameters for the reward function.

	**ω_*v*_**	**ω_*E*_**	**ω_*l*_**	**ω_*s*_**	***C*_1_**	***C*_2_**	***C*_3_**
LP1	[0, 1.0]	0.06	0.2	0	1.0	1.0	0.5
LP2	[0, 1.0]	0.2	0.2	0.15	1.0	1.0	0.5
LP3	[0, 2.5]	0.2	0.2	0.15	1.0	1.0	0.5

#### 2.3.2. Learning scheme

This study aimed to generate multimodal gait patterns by learning appropriate actuator outputs according to the input command ω_*v*_ values, which are the weight coefficients of the velocity term in the reward function in Equation (2). ω_*v*_ determines the relative significance of the forward velocity in the reward. For instance, assuming that the model moves in ẋ = 2.0 *m*/*s* again, the velocity term in the reward function, i.e., ω_*v*_ẋ, adds 5.0 to the reward when ω_*v*_ = 2.5 and 0 when ω_*v*_ = 0. Thus, a higher ω_*v*_ helps an agent learn high-speed locomotion. An agent was trained by changing the ω_*v*_ value per epoch, which was 1,000 time steps in this study. The bipedal model was set back to the initial state when an epoch ended or when the model fell, i.e., the model tilted forward by more than 80 degrees. We add ω_*v*_ to the state variables *s*_*t*_: $s_{t}=\left\{ẋ,\theta ,\overset{∙}{\theta },h_{r},{ḣ}_{r},h_{l},{ḣ}_{l},\varphi_{r},{\overset{∙}{\varphi }}_{r},\varphi_{l},{\overset{∙}{\varphi }}_{l},{ḋ}_{r},{ḋ}_{l},\omega_{v}\right\}\in {\mathbb{R}}^{14}$.

Some hyperparameters for the reward function, including the range of ω_*v*_, were changed depending on the learning phases inspired by curriculum learning (Brendan et al., 2020). Learning was divided into three phases: “Learning Phase 1 (LP1),” “Learning Phase 2 (LP2),” and “Learning Phase 3 (LP3),” where the agent was trained using different parameters, as shown in Table 2. In LP1, we set ω_*E*_ = 0.06 and ω_*s*_ = 0, and a relatively small range of ω_*v*_∈[0, 1.0]. In this phase, the agent was encouraged to learn a forward-motion movement by swinging its legs. In LP2, ω_*E*_ was increased to 0.2 and ω_*s*_ to 0.15 to penalize large variations in the total energy and nonsymmetric movement. In LP3, the range of ω_*v*_∈[0, 2.5] was expanded, such that an agent can learn a wide range of velocities.

### 2.4. Simulation environment

MuJoCo (Todorov et al., 2012) was used as the physics simulation engine. MuJoCo provides a fast and accurate simulation environment. Thus, they are widely used in the fields of robotics and biomechanics. The MuJoCo simulator can reproduce complex dynamic systems with many contact points.

## 3. Simulation results

The hyperparameters for the SAC were set to α = 0.2 and γ = 0.99. The neural networks used in the actor and critic had two hidden layers of 100 nodes. We empirically selected a small number of hidden layers and nodes for efficient learning. The actor accepts the current state $s_{t}\in {\mathbb{R}}^{14}$ and outputs the actuator signals $a_{t}\in {\mathbb{R}}^{2}$. The critic accepts the current state $s_{t}\in {\mathbb{R}}^{14}$ and action $a_{t}\in {\mathbb{R}}^{2}$. Reinforcement learning was run for ten million steps; LP1 was performed in the initial 500 thousand time steps, LP2 in the next 2.5 million time steps, and LP3 in the remaining 7 million time steps (Table 2). At each time step, the policy was updated by using a replay buffer with the recent one million samples, with a mini-batch size of 256. The maximum length of each episode was set to 1,000. The time required for training was approximately 2 days on a Lenovo ThinkPad E470 20H2S04L00. The generated gait can be seen in the video available as Supplementary material.

### 3.1. Generated steady gaits

**Figure 3:**
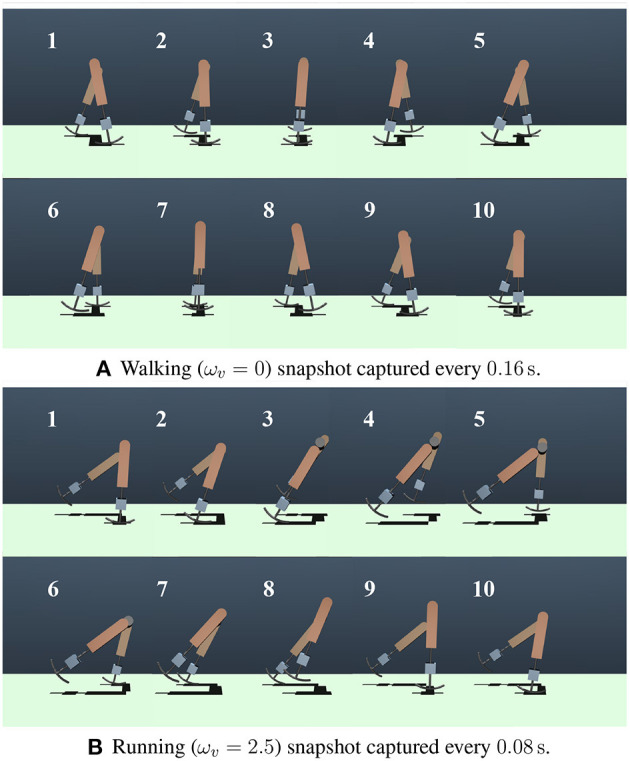
Snapshots of the generated gaits. **(A)** Walking (ω_*v*_ = 0) snapshot captured every 0.16s. **(B)** Running (ω_*v*_ = 2.5) snapshot captured every 0.08s.

**Figure 4:**
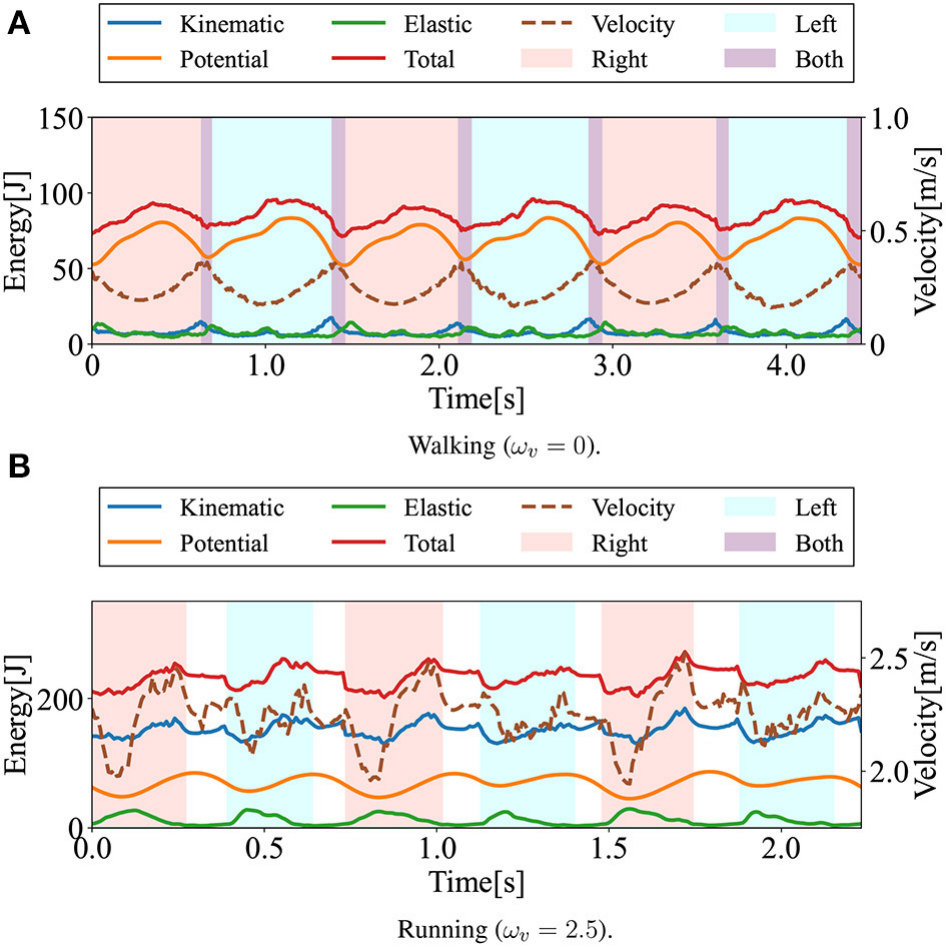
Time evolution of energies during walking and running: kinetic (blue), potential (orange), elastic (green), and total (red) energies. The dotted lines represent locomotion velocity. The pink and sky blue colored areas represent the right and left single stance phase, respectively. The purple and white colored areas represent the double stance (DS) phase and the flight (F) phase. **(A)** Walking (ω_*v*_ = 0). **(B)** Running (ω_*v*_ = 2.5).

In this section, the gaits generated in the numerical simulations are presented. Figure 3 shows the snapshots of the generated steady gaits for Figure 3A ω_*v*_ = 0 and Figure 3B ω_*v*_ = 2.5. Figure 4 shows the time evolution of the kinematic, potential, and elastic energies for both cases; the upper and lower graphs correspond to the cases in Figures 3A, B, respectively. Walking and running are defined as gaits that progress through a periodic double stance (DS) phase when both legs are on the ground, and through periodic flight (F) phases when both legs are in the air, respectively, Diedrich and Warren (1995) and Alexander (1996). The upper graph of Figure 4 indicates the periodic DS phases (purple areas), whereas the lower graph indicates the periodic F phases (white areas). The results indicate that the gait generated for ω_*v*_ = 0 exhibited an out-of-phase relationship between the kinematic and potential energies, whereas the gait generated for ω_*v*_ = 2.5 exhibited an in-phase relationship in the single stance phase. In human walking, the kinetic and potential energies exhibit an out-of-phase relationship, with potential energy being the maximum in the mid-stance phase and kinetic energy being the maximum in the DS phase (Cavagna et al., 1976, 2000; Cavagna and Legramandi, 2020). In human running, the kinetic and potential energies change in-phase during the single stance phase, both decreasing from initial contact to the mid-stance phase and increasing from the mid- to late-stance phase (Cavagna et al., 1976; Cavagna, 2006). Therefore, the phase relationship between the kinematic and potential energies in the generated gaits reflects the features of the human gaits. Furthermore, considering that the bipedal model weight (40kg) is roughly half the weight of an adult, the magnitude of kinetic energy in both walking and running is in good agreement with the human measurement data (Cavagna, 2006; Cavagna and Legramandi, 2020).

**Figure 5:**
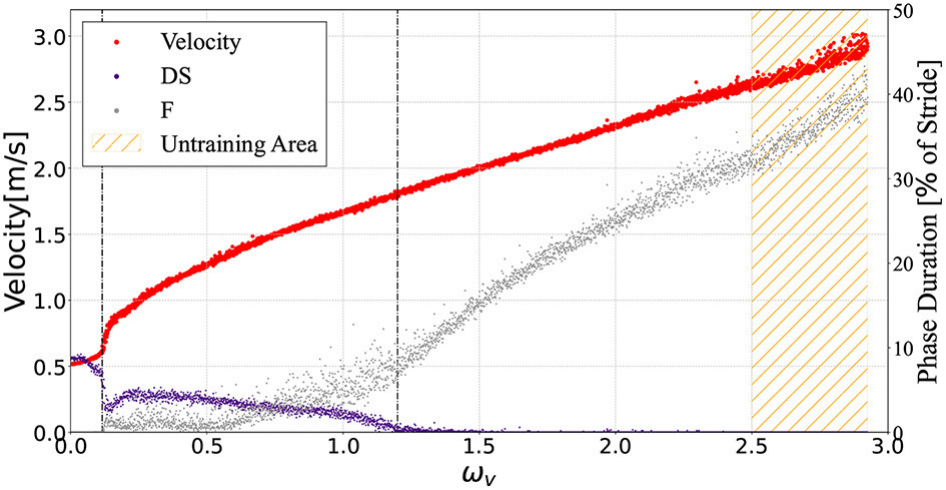
The average model velocities for the input ω_*v*_. The purple and gray points denote the ratio of the DS and F phase in the gait cycle respectively. The controller generated steady gaits in the bipedal model for 0 ≤ ω_*v*_ ≤ 2.93, even though we did not train with 2.5 ≤ ω_*v*_ ≤ 2.93 (orange hatched area). This graph shows the plots when the input command ω_*v*_ was given in the range of 0.0–2.93 in 0.01 intervals. For each ω_*v*_, the biped model locomoted for a distance of 30 m from the initial state.

Figure 5 illustrates the average velocities and the DS and F phase ratios of a single gait cycle in response to the value ω_*v*_∈[0, 2.93]. The controller generated a running gait for parameters ω_*v*_∈[2.5, 2.93] outside the training range ω_*v*_∈[0, 2.5]. The speed range observed in this bipedal model was 0.51 ≤ ẋ ≤ 3.02m/s. A periodic DS phase was observed for 0 ≤ ω_*v*_ ≤ 0.1, while an F phase was not observed. In the range ω_*v*_≃0.1, the ratio of the DS phase suddenly decreased; then, for 0.1 ≤ ω_*v*_ ≤ 1.2, transient gait patterns with nonperiodic DS and F phases were found. For ω_*v*_≥1.2, neither DS nor periodic F phases were found.

**Figure 6:**
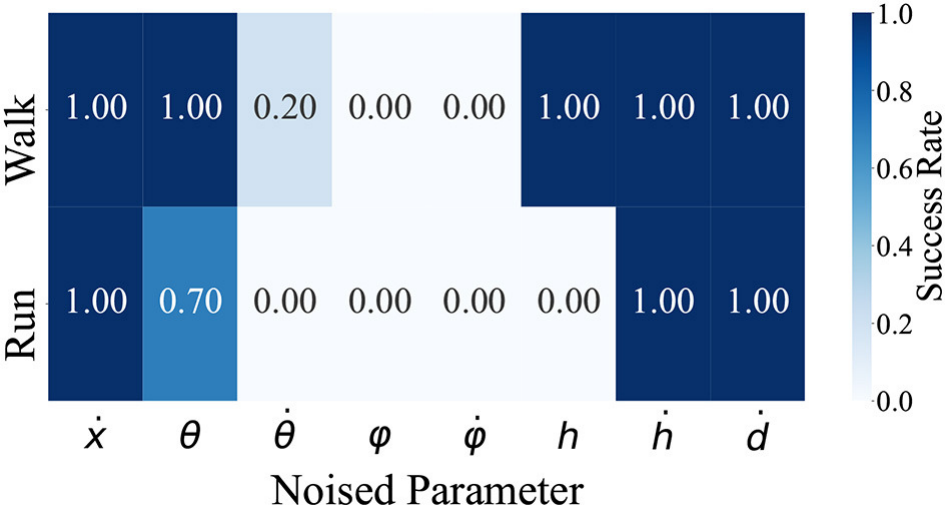
The rate of steady gait was maintained when noise was applied. $ẋ,\theta ,\overset{∙}{\theta },\varphi ,\overset{∙}{\varphi },h,ḣ,ḋ$ are the input parameters for the controller. For each of these seven states, we added Gaussian noise during steady walking and running and checked whether the bipedal model maintains the gaits. We conducted 10 locomotion trials for each condition and judged the success case when the bipedal model was able to move 10m in the walking gait and 20m in the running gait after noise was applied.

We identified the parameters essential for the steady walking and/or running gaits by adding noise to the model state inputs for the controller. As mentioned, the observed model states for the controller comprises the model velocity ẋ, hip segment orientation θ and its change rate $\overset{∙}{\theta }$, leg angles φ and their change rate $\overset{∙}{\varphi }$, displacements of the thigh segments *h* and their change rate ḣ, and leg contraction rates ḋ. For each of these seven observation states, we examined whether steady walking (ω_*v*_ = 0) and running (ω_*v*_ = 2.5) could be maintained when Gaussian noise with the mean 0 and the standard deviation σ was added. Since each observation state has a different scale, calculating the noise with the same standard deviation is not reasonable. Therefore, we measured the time evolution of each observation state during steady walking (ω_*v*_ = 0) and running (ω_*v*_ = 2.5) and calculated standard deviations, then set these values as the standard deviation of the noise; the detailed values are shown in Table 3. Figure 6 shows the noised parameters; the rates of gait were maintained (i.e., the model did not all down). Both generated walking and running are susceptible to noise at $\overset{∙}{\theta }$, φ, and $\overset{∙}{\varphi }$. Moreover, in the running gait, the bipedal model was vulnerable to noise at *h* and θ.

**Table 3:** Standard deviation of gaussian noise.

**σ_*walkẋ*_**	**0.3476 [m/s]**	**σ_*runẋ*_**	**0.6743 [m/s]**
σ_*walkθ*_	0.2757 [rad]	σ_*runθ*_	0.3053 [rad]
$\sigma_{walk\overset{∙}{\theta }}$	0.7986 [rad/s]	$\sigma_{run\overset{∙}{\theta }}$	1.0773 [rad/s]
σ_*walkh*_	0.1942 [m]	σ_*runh*_	0.3007 [m]
σ_*walkḣ*_	0.5596 [m/s]	σ_*runḣ*_	0.9818 [m/s]
σ_*walkφ*_	0.4433 [rad]	σ_*runφ*_	0.5628 [rad]
$\sigma_{walk\overset{∙}{\varphi }}$	0.9192 [rad/s]	$\sigma_{run\overset{∙}{\varphi }}$	1.664 [rad/s]
σ_*walkḋ*_	0.3110 [m/s]	σ_*runḋ*_	0.4349 [m/s]

Because the scales of the observation states were not consistent, the standard deviation values of the noise depended on the states. We measured the time evolution of each observation state during steady walking (ω_*v*_ = 0) and running (ω_*v*_ = 2.5) and calculated standard deviations. Then we set these values as the standard deviations of the Gaussian noise.

### 3.2. Generated gaits during training

**Figure 7:**
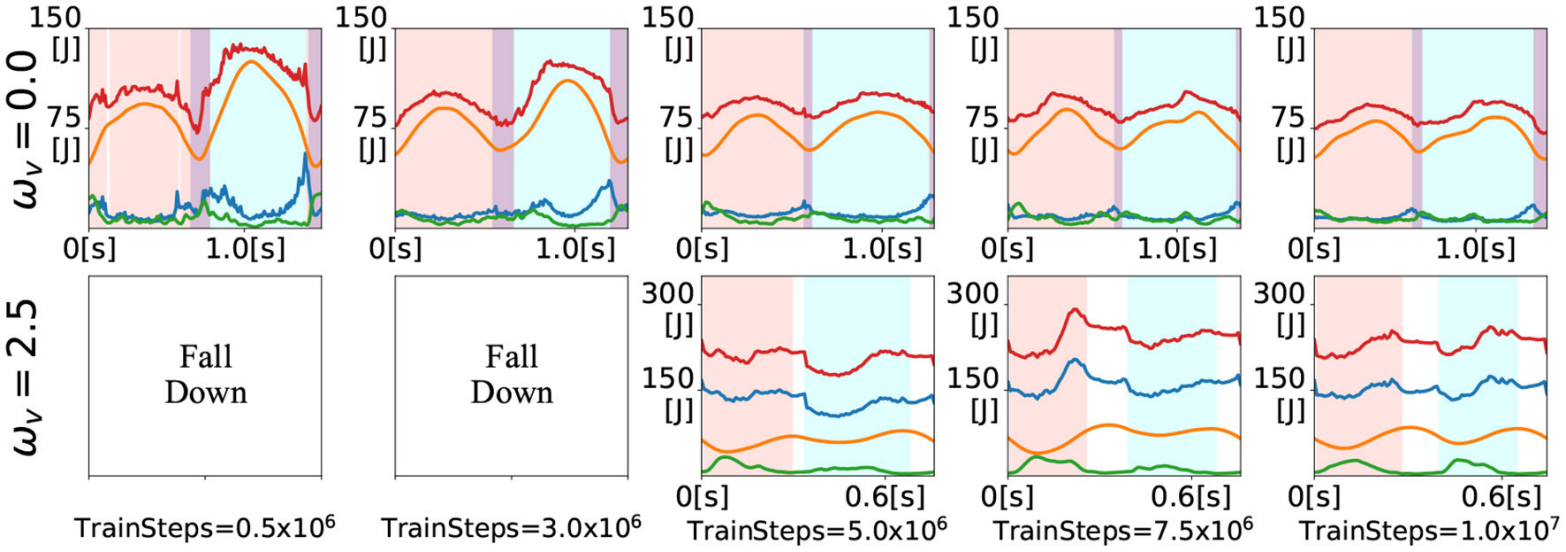
Time evolution of energies for the inputs ω_*v*_ = 0 and ω_*v*_ = 2.5 during training. The colored lines denote the kinematic (blue), potential (orange), elastic (green), and total (red) energies. The shaded areas denote the right single stance phase (pink), left single stance phase (sky blue), DS phase (purple), and F phase.

In this section, we show the gaits generated during training for the inputs ω = 0 and ω = 2.5. Figure 7 shows the time evolution of the energies at 500 thousand (at the end of LP1), 3 million (at the end of LP2), 5 million, 7.5 million, and 10 million (at the end of training) training time steps. The upper figures show the generated gaits for ω = 0 and the lower figures show the generated gaits for ω = 2.5. For the input ω = 0, the walking gait was already generated at the 500 thousand training time steps. Thereafter, the total energy variation reduced as the training progressed, and at the middle of the training (i.e., 5 million training time steps), the time evolution of the energies was almost the same as that at the end of the training. For the input ω = 2.5, the bipedal model kept falling down until 3 million training time steps. The running gait appeared at the middle of the training. However, it was asymmetric, as evidenced by the relatively short DS phase (white area) after the right single stance phase (pink area). As training progressed, the duration of the DS phase became almost the same. In addition, elastic energy (green) began to be used during the left single stance phase (sky blue area). The gaits generated during training can be seen in the video available as Supplementary material.

### 3.3. Energetics

The speed range obtained in this bipedal model was 0.51 ≤ ẋ ≤ 3.02m/s, and the Froude number (*Fr*) was 0.18 ≤ *Fr* ≤ 1.08, using $Fr=\frac{ẋ}{\sqrt{gl}}$. In this study, we investigated the energetics of the generated gaits, which can be divided into three gait patterns according to the measured locomotion velocity shown in Figure 5: walking gait for 0.51 ≤ ẋ ≤ 0.63m/s, transient gait for 0.63 ≤ ẋ ≤ 1.81m/s, and running gait for ẋ≥1.81m/s. As mentioned before, walking and running gaits are gait patterns with periodic DS and F phases, respectively. Transient gait is defined as a gait pattern with both DS and F phases; however, these phases are not periodic. Here, we numerically evaluated the energy efficiency using the cost of transport (CoT) (Ruina et al., 2005), which is described by the following equation:


(8) $$\begin{array}{l} CoT=\frac{\Delta W}{mg\Delta x}=\frac{1}{mg\Delta x}\underset{j\in r,l}{\sum }\overset{t_{end}}{\underset{t_{0}}{\int }}\max\left(F_{a,j}\left(t\right){ḣ}_{j}\left(t\right),0\right)dt, \end{array}$$

**Figure 8:**
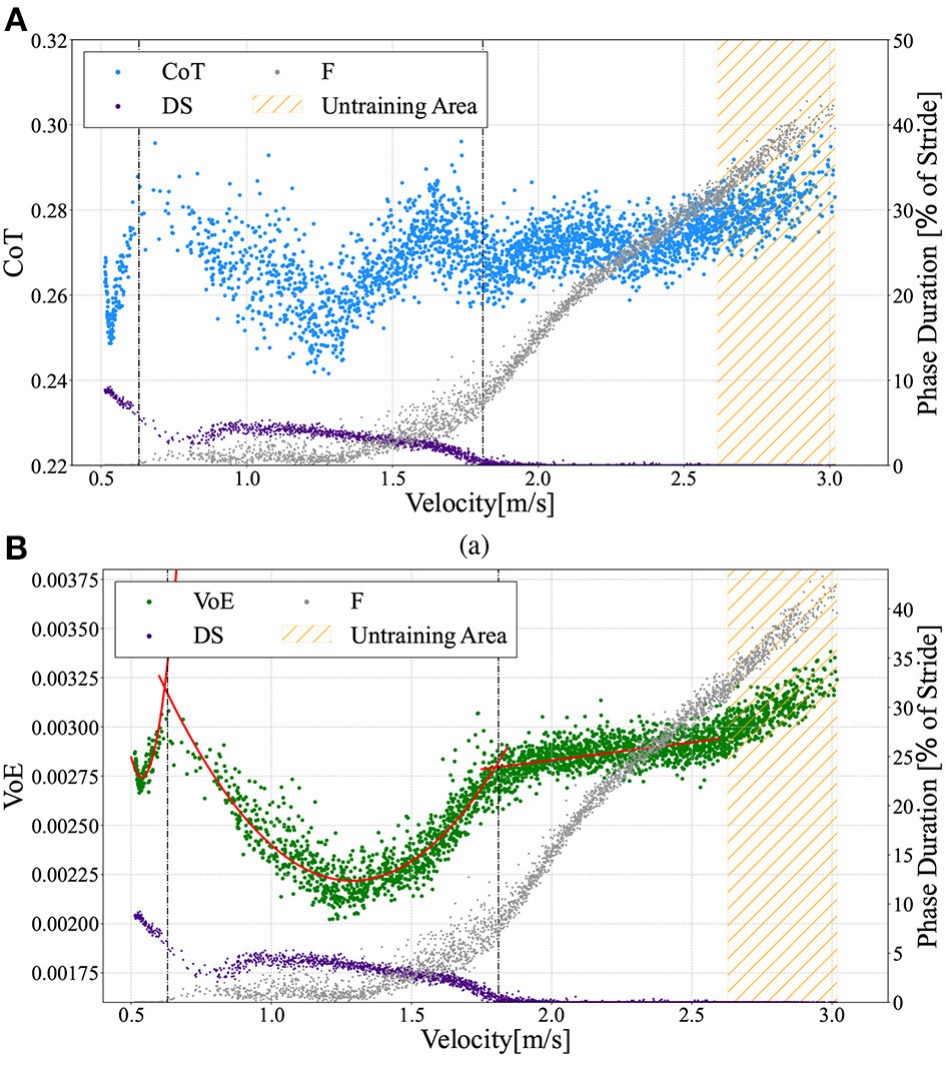
**(A)** Cost of Transport (CoT) vs. measured velocities ẋ. The scatter graphs here include data from a 10 to 30 m distance locomotion in the ω_*v*_ range from 0.0 to 2.93 in 0.01 intervals, similar to Figure 5. **(B)** Variation of Energy (VoE) vs. measured velocities ẋ. Gaits can be divided into three patterns: (1) walking 0.51 ≤ ẋ ≤ 0.63m/s, (2) transient gait 0.63 ≤ ẋ ≤ 1.81m/s, and (3) running gait ẋ≥1.81m/s. The vertical dotted lines describe the borders at ẋ = 0.63m/s (left) and ẋ = 1.81m/s (right). The VoE for walking gait and transient gait can be approximated by quadratic curves (red lines) of $VoE_{w}=0.0694{ẋ}^{2}-0.075ẋ+0.0229$ and $VoE_{t}=0.0022{ẋ}^{2}-0.0058ẋ+0.0059$, respectively. For the running gait mode, a straight line represents the function *VoE*_*r*_ = 0.00018ẋ+0.00246.

Where Δ*W* denotes the total energy consumption, *m* is the model mass, *g* is the acceleration due to gravity, Δ*x* is the distance traveled, *F*_*a, j*_(*t*) is the force of each actuator, and ḣ_*j*_(*t*) denotes the displacement velocity of each thigh segment. Figure 8A shows the CoT profile as a function of the measured velocity. A lower CoT value indicates energy-efficient locomotion.

Moreover, we defined the variation of energy (VoE), which is the time integral of the total energy variation, as follows:


(9) $$\begin{array}{l} VoE=\frac{\Delta E}{mg\Delta x}=\frac{1}{mg\Delta x}\overset{t_{end}}{\underset{t_{0}}{\int }}|E_{t}-E_{t-1}|dt, \end{array}$$

Where Δ*E* is the sum of the total energy changes from *t*_0_ to *t*_*end*_. For a lower CoT (i.e., energy-efficient) gait, the VoE also tends to be lower because the energy injected from the actuators and the energy lost at touchdown are small and the total energy remains constant. Hence, there is a high correlation between the VoE and CoT. Figure 8B illustrates the relationship between the measured locomotion velocities and the VoE. A comparison of Figures 8A, B shows that the change in CoT against the measured velocity demonstrates a trend similar to that of VoE except for the range of 1.7 ≤ ẋ ≤ 1.9m/s, for which the CoT shows a downward convex trajectory. We can see that the trajectory of the VoE can be clearly divided for each of the three gait patterns. The VoE for the walking gait and transient gait can be approximated by different quadratic curves (red lines), as shown in Figure 8B. The VoE for the running gait is a linear line proportional to the velocity.

### 3.4. Adaptability to environmental changes

**Figure 9:**
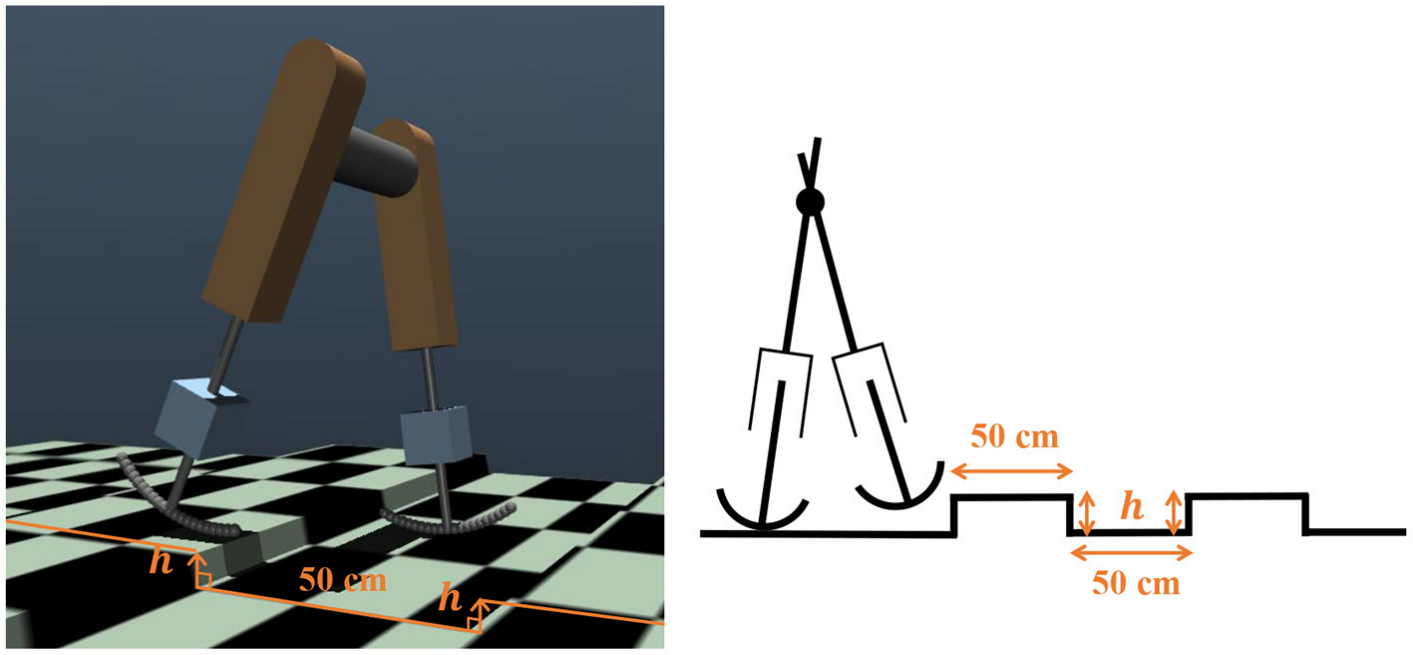
Environmental changes introduced using steps in a simulation environment and its diagram.

We investigated whether the trained controller could adapt to environmental changes. For this purpose, we set up a stepped environment, where steps of a constant height *h* appeared every 50 cm, as shown in Figure 9. We verified the steady walking (ω_*v*_ = 0) and steady running (ω_*v*_ = 2.5) performance in this environment. We judged the success case as the condition in which the bipedal model successfully covered a distance of 30m. The bipedal model can move without falling down up to *h* = 1.0cm in the walking gait and *h* = 0.8cm in the running gait. The attached movie shows the bipedal model walking and running in a changing environment.

### 3.5. Gait transitions

**Figure 10:**
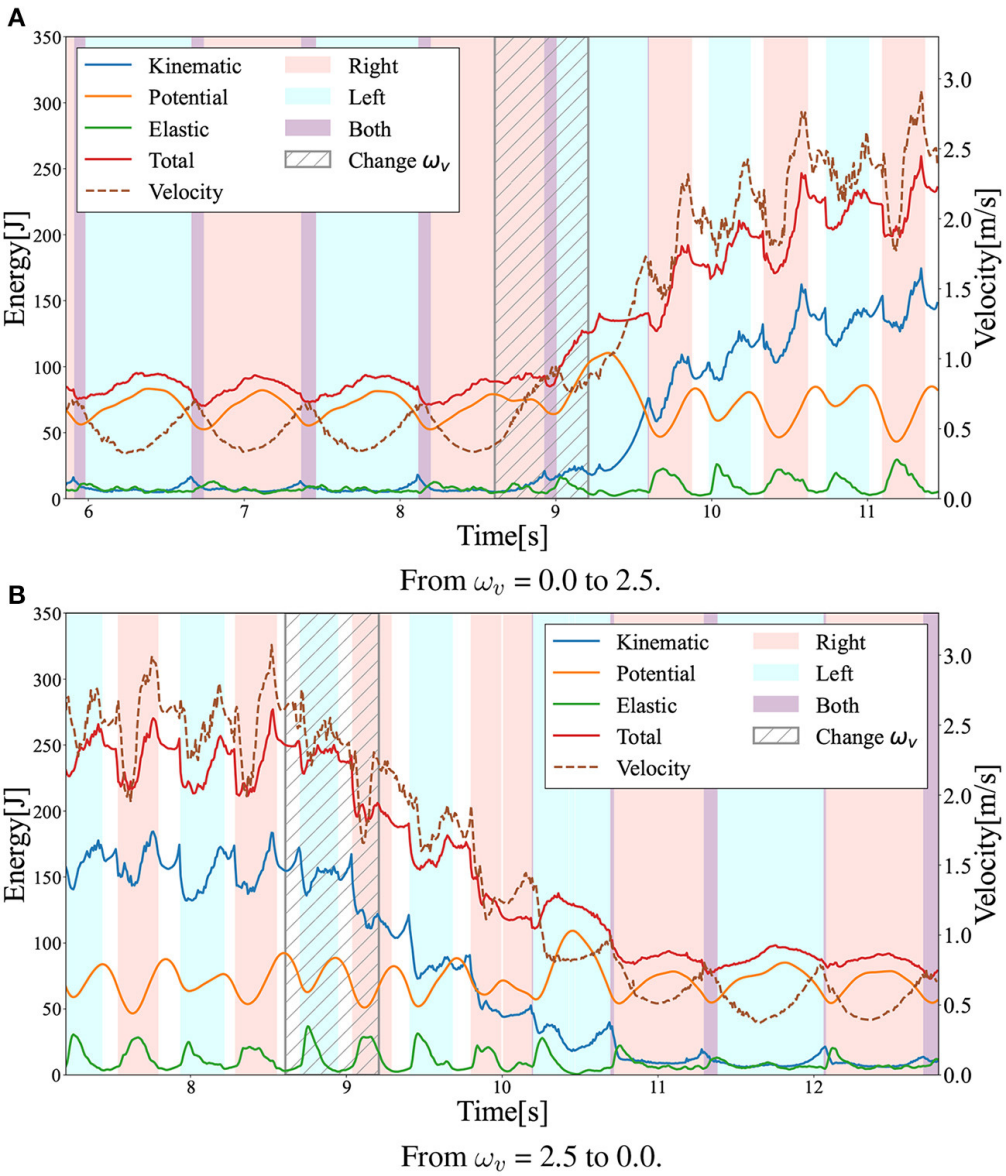
Gait transition between walking and running with changes in the ω_*v*_ value. The graph shows the time evolution of kinetic (blue), potential (orange), elastic (green), and total (red) energies during the gait transition. The dotted lines represent locomotion velocity. The pink and sky blue areas represent the right and left single stance phase, respectively. The purple and white areas represent the double stance (DS) and flight (F) phases. We linearly changed the ω_*v*_ in the shaded period. **(A)** From ω_*v*_ = 0.0 to 2.5. **(B)** From ω_*v*_ = 2.5 to 0.0.

To evaluate the gait transition ability of the acquired policy, the gait pattern was observed under a change in the command value ω_*v*_ during locomotion. In this simulation, the input ω_*v*_ was linearly increased from 0.0 to 2.5 at 0.6 s in Figure 10A, whereas ω_*v*_ was linearly decreased from 2.5 to 0.0 at 0.6 s, as shown in Figure 10B. For both changes in ω_*v*_, the acquired policy could successfully achieve gait transition. Note that the transition from walking to running was successfully completed in less than two steps, whereas the transition from running to walking required approximately four steps. Moreover, not only single transitions such as walk-to-run and run-to-walk, but also multiple transitions such as walk-to-run-to-walk were achieved.

**Figure 11:**
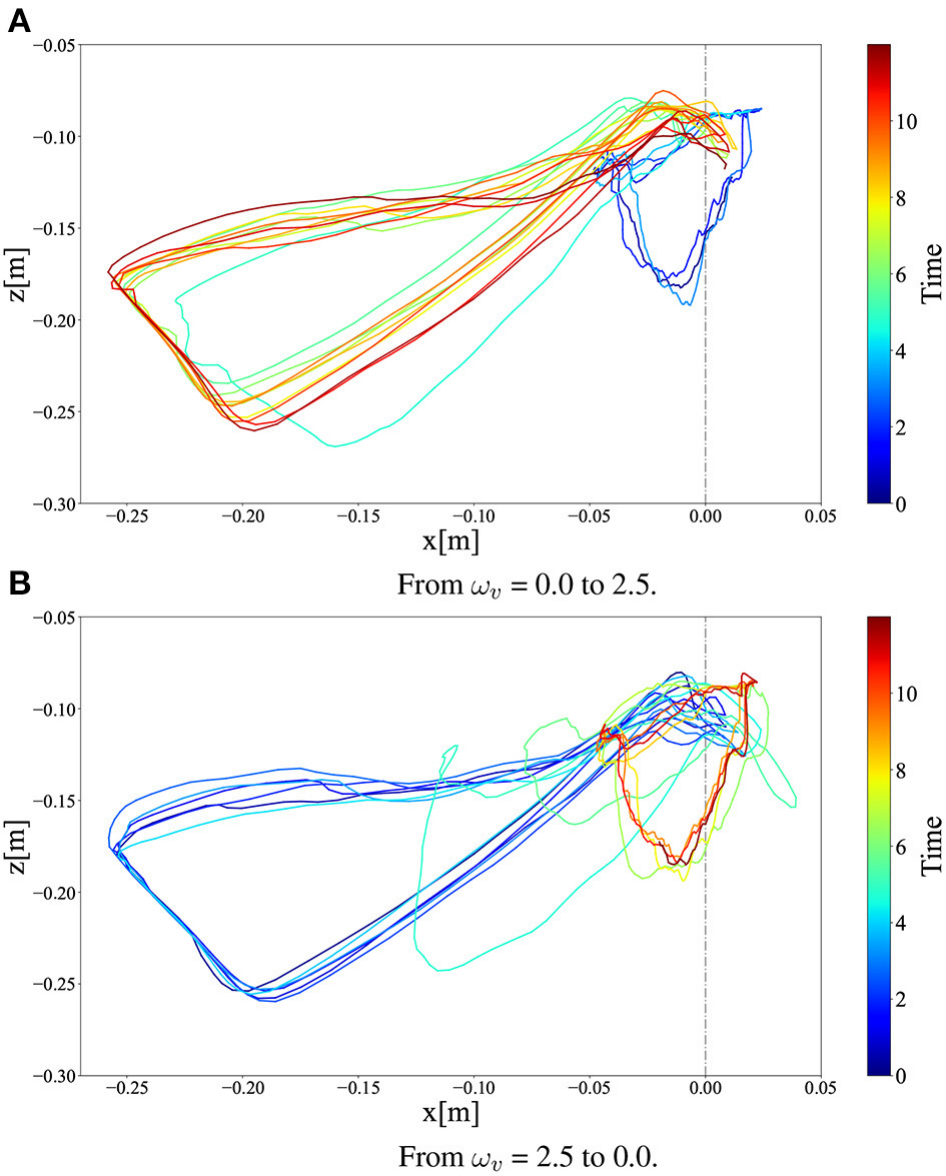
*x*–*z* CoM trajectories of the right thigh segment in **(A)** walk-to-run transition and **(B)** run-to-walk transition for 12 s (before and after the ω_*v*_ change). In the figures, we linearly changed ω_*v*_ at 4 s.

Figure 11 illustrates the *x*–*z* CoM trajectories of the right thigh segment with respect to the hip joint position (Figure 1) for 12 s (before and after the ω_*v*_ change). As shown in Figure 11, ω_*v*_ changed linearly from 0 to 2.5 in Figure 11A and from 2.5 to 0 in Figure 11B in 0.6 s. In the steady-state motion, it can be observed that walking and running converge to different limit cycles, smaller ones to the right and larger ones to the left, respectively. This limit cycle analysis also confirmed that the transition from walking to running was achieved smoothly and stably, while that from running to walking required more time steps.

**Figure 12:**
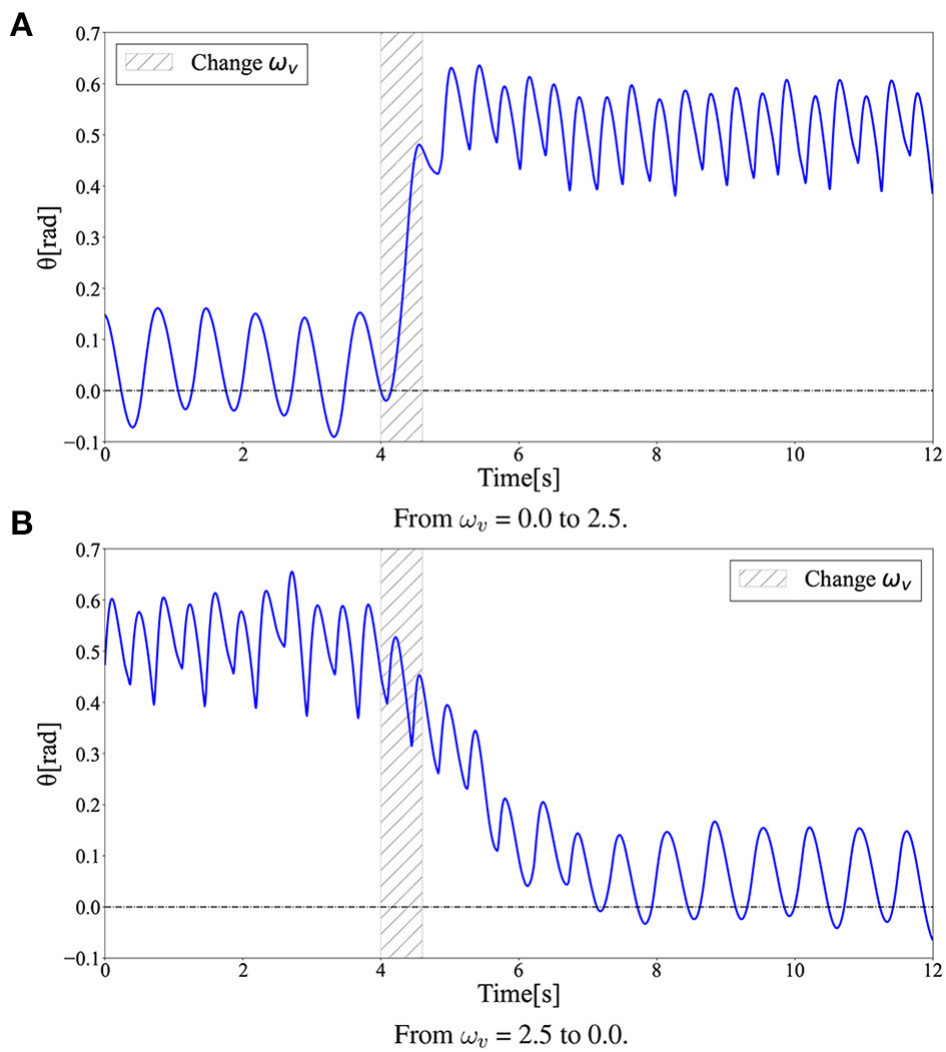
Hip segment orientation (θ) during gait transition. The gait patterns generated by the learned policy show that the bipedal model maintained a relatively perpendicular posture to the ground (θ≈0) when walking, while the model moved with a forward leaning posture (θ>0) when running. **(A)** From ω_*v*_ = 0.0 to 2.5. **(B)** From ω_*v*_ = 2.5 to 0.0.

Figure 12 shows the time evolution of the hip segment orientation θ (see Figure 1) during gait transitions. The gait patterns generated by the learned policy show that the bipedal model maintained a relatively perpendicular posture to the ground (θ≈0) when walking, whereas the model moved with a forward leaning posture (θ>0) when running. This can also be observed in the snapshots of the generated gaits (Figure 3). The difference between Figures 3A, B in the gait transition duration may be due to the difference in body posture during walking and running: the transition from upright walking to leaning forward running is physically easy, whereas the transition process from leaning forward to upright walking requires the posture to return to upright, which requires more energy; thus, the physical state transition takes more time steps.

### 3.6. Comparison with partially modified reward function and learning scheme

**Figure 13:**
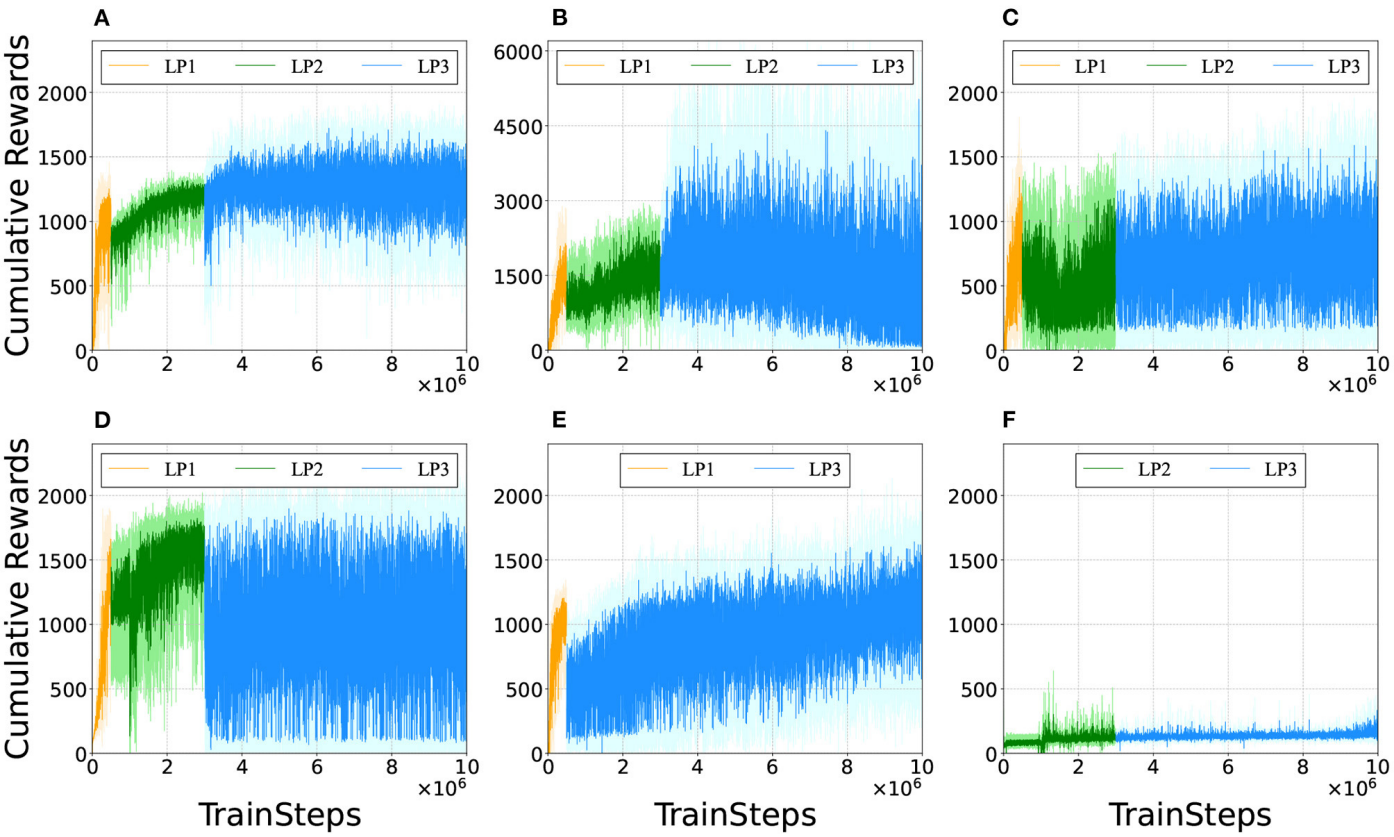
Comparison of learning curves. We evaluated the policies every 1,000 training time steps by testing the performance for one episode (the maximum length being 1,000 time steps). **(A)** Proposed method. **(B)** Excluding 1/(1+ω_*v*_) from Equation (2), i.e., the reward function was changed to *r*(*s*_*t*_, *a*_*t*_) = −ω_*E*_|*E*_*t*_−*E*_*t*−1_|+ω_*v*_ẋ+*f*_*forward*_+*f*_*alive*_+*f*_*support*_. 1/(1+ω_*v*_) normalizes the value among the rewards that vary widely due to velocity term ω_*v*_ẋ. **(C)** The range of ω_*v*_ in LP1 and LP2 was set to [0,2.5]. The agent was trained to learn appropriate outputs for ω_*v*_∈[0, 2.5] from the beginning. **(D)** The range of ω_*v*_ in LP1 and LP2 was changed to [1.5,2.5]. The agent was trained to learn appropriate outputs for larger ω_*v*_ first than smaller ω_*v*_. **(E)** Without LP2. The reward function was set with LP1 parameters in the initial 500 thousand training time steps and with LP3 parameters in the rest of 9.5 million training time steps. **(F)** Without LP1. The reward function was set with LP2 parameters in the initial 3 million training time steps and with LP3 parameters in the rest of 7 million training time steps.

To investigate the factors contributing to effective multimodal locomotion learning, the agents were trained with partially modified reward functions. Figure 13 shows the representative learning curves for Figure 13A our proposed method; Figure 13B excluding 1(1+ω_*v*_) from Equation (2) not to reduce inter-reward variability; Figure 13C the range of ω_*v*_ in LP1 and LP2 was set to [0,2.5]; Figure 13D the range of ω_*v*_ in LP1 and LP2 was set to [1.5,2.5]; Figure 13E trained without LP2; and Figure 13F trained without LP1. In these figures, cumulative rewards close to 0 imply that the bipedal model did not move forward or immediately fell. Note that there was a significant amount of variance in the cumulative rewards even after the completion of training. This was because the reward function calculated widely different values depending on the given ω_*v*_∈[0, 2.5], which changed randomly during the training.

Figure 13B shows that the cumulative rewards gradually dropped to 0 after training for 6 million time steps. It was observed that for relatively small ω_*v*_, the bipedal model exhibited steady locomotion by the 6-million-time-step training; however, it became unstable around 7 million time steps and fell immediately after 8 million time steps. Note that the results in Figure 13B were obtained by excluding 1/(1+ω_*v*_) from the reward function; hence the range of cumulative rewards is different from that in the other subfigures.

In Figure 13C, ω_*v*_ was fixed to [0, 2.5] through training, and the agent acquired only walking gaits and fell after a few steps for a relatively large ω_*v*_. In Figure 13D, the value of ω_*v*_ in LP1 and LP2 was increased; the agent obtained only running gait; they could not find walking gait and fell immediately for a relatively small ω_*v*_. In Figure 13E, it can be observed that training the agent without LP2 took more time steps to learn the appropriate outputs for ω_*v*_ compared to Figure 13A. From Figure 13F, the agent trained without LP1 failed to find control solutions for steady locomotion; it fell from the start position. A comparison of these results suggests that LP1 is an essential phase for learning the basic gait pattern from an unlearned state, while LP2 is necessary for learning a more stable gait pattern from the initially acquired gait pattern.

## 4. Discussion

This study demonstrated that a trained controller can generate walking, running, and transient gaits in a bipedal model using passive dynamics. We also found that the trained controller was adaptable to environmental changes during steady walking and running. Moreover, the bipedal model with the trained controller exhibited gait transitions by changing a single parameter ω_*v*_. These results suggest that DRL can be applied to generate multimodal bipedal locomotion using passive dynamics. Additionally, the energy efficiency of the locomotion generated by the policy acquired through DRL was verified, including the reliability of learning using curriculum learning (Brendan et al., 2020), i.e., the parameter planning for the settings of the weights for the rewards during the learning process.

Here, Equation (2) was used as the reward function. The first term −ω_1_|*E*_*t*_−*E*_*t*−1_| significantly contributed to the generation of continuous locomotion in the bipedal model. Before this term was included, no control solutions were found for walking and running, and the bipedal model exhibited acyclic, velocity-unstable, and fall-prone movements. Humans generate walking and running motions with small energy fluctuations by effectively using their body dynamics. During walking, humans behave like an inverted pendulum and exchange kinematic and potential energy to conserve mechanical energy. During running, humans store potential and kinematic energy in the spring elements of their bodies to reduce energy loss, resulting in less energy fluctuations. From Figure 4, it can be observed that the presented bipedal model employed the same strategy for the body dynamics observed in humans. An out-of-phase relationship was observed between kinetic and potential energy in the walking gait. In the running gait, during the initial single stance phases, elastic energy increased sharply, whereas both the kinematic and potential energies fell. Subsequently, the elastic energy gradually decreased with kinematic and potential energy increments. We consider that the first term in the reward function contributes to achieving walking and running in the bipedal model because this term helps the model mimic the energy variation in time, similar to how humans generate walking and running motions.

The constant parameters in the reward function, ω_*E*_, ω_*l*_, and *C*_3_, need to be set carefully. When ω_*E*_, which determines the penalty for the total energy variation, is set to a small value, the generated gaits appear awkward, as shown in the time evolution of energies for five thousand samples in Figure 7. Additionally, when ω_*E*_ is set to a large value from the beginning, no gait is generated, as shown in Figure 13F, because of the excessive penalty for the movement. We consider the approach of acquiring the basic gait pattern with a small penalty for energy in the initial phase, and acquiring the learning movements with a large penalty for energy as the training progresses, as effective in generating efficient gaits. When either ω_*l*_ or *C*_3_ was set to a very small value, the agent did not tend to acquire a steady gait. However, when either ω_*l*_ or *C*_3_ was set to a very large value, the trained controller exhibited a high kicking gait. *f*_*leg*_ ensures efficient learning but needs to be set carefully.

Figure 6 shows that although the generated walking was robust to the noise applied to the observed displacements of the thigh segments *h*, the generated running was extremely susceptible to the noise applied to *h*. In the IP model, which is a simple conceptual walking model, the stance leg is rigid and its length remains constant. However, in the SLIP model, which is a simple conceptual running model, the stance leg is represented as a spring and the leg length varies. Therefore, it can be inferred that the parameter *h*, which indicates the length of the leg of the biped model, is an important parameter in generating the running gait.

The gait speed obtained using the bipedal model was 0.51 ≤ ẋ ≤ 3.02m/s. The proposed bipedal model has a wide range of speeds. These results indicate that by utilizing body dynamics effectively, the bipedal model can move over a wide range of speeds. It should be emphasized that the hip joints of the proposed bipedal model are completely passive. The actuation of the hip joint has a significant effect on locomotion velocity (Dzeladini et al., 2014; Bailey et al., 2017). For example, humans change the activity pattern of the muscles around the hip joint, e.g., gluteus maximus and rectus femoris, depending on their speed (Cappellini et al., 2006). To move faster, the proposed bipedal model leaned its body forward and vigorously moved its legs up and down to obtain a larger propulsion force in the stance phase.

Interestingly, the required time for the transition to the other limit cycles of the gait was different between the walk-to-run transition and run-to-walk transition: walk-to-run was faster, whereas run-to-walk required additional time steps. Moreover, Figure 10A shows that the kinetic energy increases with increase in ω_*v*_ in the walk-to-run transition, despite the time delay between the start of decrease in ω_*v*_ and decrease in the kinetic energy peak in the run-to-walk transition, as shown in Figure 10B. These observations can be attributed to hysteresis, i.e., the transition process dynamics depend on the previous gait pattern, which is attributed to the transition between different attractors (Diedrich and Warren, 1995). As shown in Figure 12, the differences in body posture owing to gait dynamics were confirmed. We assume that these differences in the attractor dynamics owing to the gait characteristics resulted in differences in the convergence process.

Comparing Figures 13A, B, it can be observed that reducing inter-reward variability improved the reliability of policy learning in multimodal locomotion. This is because the reward function without 1/(1+ω_*v*_) calculates widely different values, depending on the given ω_*v*_ as mentioned in designing the reward function. The agent was trained with a bias toward relatively high ω_*v*_ to obtain higher cumulative rewards. This fact suggests that the variation among rewards should be as small as possible during the policy-learning process to eliminate bias. The results also indicated that the ω_*v*_ range of the training progress affected the performance of the learned controller. It was also observed that the model failed to learn the walking gait when the value of ω_*v*_ was set to high in the initial learning stage (Figure 13D). Additionally, learning locomotion with a wide range of ω_*v*_ from the beginning makes training difficult (Figures 13C, E). These facts suggest that training to learn low-speed locomotion initially and then gradually transferring to high-speed locomotion is the key to effective multimodal locomotion learning.

In this study, the proposed method was validated exclusively through simulations. Therefore, building a hardware and verifying its feasibility in the real world would be of primary interest in future studies. Because the controller realized in this study is simple and is able to adapt to a new surface containing steps, we believe that the trained controller can be applied to suitable hardware. However, the gaps between the simulation environment and hardware need to be solved. A major gap is control latency. In this study, we did not consider the time delay between the sensors, controller, and actuators. In addition, as shown in Figure 6, some of the input parameters of the controller, namely, $\overset{∙}{\theta }$, φ, $\overset{∙}{\varphi }$, *h*, and θ were sensitive to noise in steady gait. Therefore, the hardware must be designed to accurately measure these parameters. Moreover, the bipedal model motion was achieved only in a forward straight line. Hence, the extension of the motion space into three dimensions and designing of a learning framework that can change the motion direction are other important issues that need to be resolved.

## 5. Conclusion

Gait generation in underactuated robots requires control solutions that can achieve stability with input from a limited number of active actuators. To reproduce multimodal locomotion, it is necessary to provide control solutions that generate motion patterns for drastically different modes in terms of dynamics, which is an extremely challenging optimization problem. Thus, multimodal locomotion using passive dynamics is an extremely challenging problem. Despite being limited to physical simulation, this study provided evidence that a bipedal model with completely passive hip joints was able to learn various motions, including walking, running, and gait transition, through DRL. Therefore, we believe that this study provides a framework that will enable walking and running with the efficient use of body morphology in bipedal robots.

## Data availability statement

The raw data supporting the conclusions of this article will be made available by the authors, without undue reservation.

## Author contributions

KK, DO, and MH conceived the study. SK developed a bipedal model and learning scheme. All the authors participated equally in the preparation of the manuscript.
